# *Ginkgo biloba* Flower Extract Accelerates the Wound Healing in Diabetic Rats by Inhibiting Inflammation and Ferroptosis

**DOI:** 10.3390/ijms27135793

**Published:** 2026-06-26

**Authors:** Xin Sun, Ruihong Li, Yingying Xu, Yuying Wang, Ziming Xia, Ying Tian, Guangjie Zhang, Sifan Liu, Min Li, Shuchen Liu

**Affiliations:** 1 School of Pharmacy, Anhui Medical University, Hefei 230032, China; sunx0102@163.com (X.S.); 15665648396@163.com (Y.X.); 2 Academy of Military Medical Sciences, Beijing 100850, China; lrh1217021@163.com (R.L.); wangyuying0829@163.com (Y.W.); zmxia22@163.com (Z.X.); tianying1977@126.com (Y.T.); zhanggj410@sina.com (G.Z.); 18146539471@163.com (S.L.)

**Keywords:** diabetic wound healing, *Ginkgo biloba* flower, ferroptosis, inflammation

## Abstract

Diabetic wounds are a common complication of diabetes mellitus (DM), with healing often impaired by ferroptosis and persistent inflammation. This study investigated the effects of *Ginkgo biloba* flower extract (GBF) on diabetic wound healing by focusing on inflammation and ferroptosis. Chemical composition analysis identified 123 compounds in GBF containing 73 active ingredients of flavonoids and 12 terpenoids. In vivo, GBF treatment significantly accelerated the wound healing process in diabetic rats, and GBF promoted epithelial regeneration and collagen deposition by increasing the expression of CD31 and VEGF. It also enhanced the formation of new blood vessels. Mechanistically, GBF could inhibit the inflammatory response by reducing the levels of tumor necrosis factor-α (TNF-α) and interleukin-6 (IL-6), and inhibit the oxidative stress-induced ferroptosis by increasing the levels of glutathione (GSH) and glutathione peroxidase 4 (GPX4). Proteomics analysis further confirmed its regulatory effects on inflammation and iron metabolism pathways. In vitro, GBF promoted the survival and migration of rat skin fibroblasts (RS1) while reducing the levels of reactive oxygen species (ROS) and Fe^2+^ in erastin-induced ferroptosis cells. In conclusion, GBF promotes diabetic wound healing by inhibiting ferroptosis and inflammation.

## 1. Introduction

Diabetes mellitus (DM) is a global chronic metabolic disorder. Recent epidemiological projections indicate that the global prevalence of diabetes will surge from 529 million in 2021 to over 1.3 billion by 2050 [1]. DM has many serious complications, among which diabetic kidney disease (DKD), one of the types of chronic kidney disease (CKD), is one of the most serious complications. In DKD, a high-glucose environment induces metabolic reprogramming of glucose, lipid and protein in proximal renal tubular epithelial cells, leading to volume overload, oxidative stress and mitochondrial dysfunction [2]. During this process, renal tubular epithelial cells undergo epithelial–mesenchymal transition (EMT) and obtain a mesenchymal fibroblast-like phenotype, which directly promotes tubular atrophy, interstitial inflammation and extracellular matrix deposition [3]. In addition, the zinc finger protein YY1 induces a positive feedback loop by up-regulating hypoxia-inducible factor-1 alpha (HIF-1α) and promoting the excessive production of mitochondrial reactive oxygen species (mROS), further aggravating the proliferation and sclerosis of glomerular mesangial cells [4]. The core pathological process in the progression of CKD to end-stage renal disease is renal fibrosis, and its molecular mechanism involves ferroptosis, oxidative stress, inflammatory response, and complex interactions of multiple signaling pathways, especially in the context of DM [5]. Diabetic wounds are another common and serious complication of DM; these wounds are strongly associated with extremely high rates of lower limb amputation and mortality. A prospective cohort study showed that the cumulative incidence of lower limb amputation within 12 months after the first occurrence of diabetic wounds was 24.2%, while the 12-month mortality rate was as high as 21.6% [6]. A recent population-based study showed a prevalence of 4.6% and an annual incidence of 2.1%, which further confirmed its high mortality rate, with a 5-year mortality rate of about 64% in patients with infectious diabetic wounds [7]. The multifactorial pathophysiological mechanisms of diabetic wounds, including chronic hyperglycemia, oxidative stress, persistent inflammation, impaired angiogenesis, and altered cellular function, further aggravate these harms [8]. Despite the devastating consequences of diabetic wounds described above, current therapeutic options remain limited. Standard care includes debridement, infection control, offloading, and revascularization when indicated, but these approaches often fail to achieve timely healing in a significant proportion of patients [9,10]. Effective strategies to accelerate the healing of diabetic wounds, control infection, prevent tissue necrosis, and reduce amputation risk remain unmet clinical and scientific challenges, primarily due to the incomplete elucidation of diabetic wound pathogenesis and the lack of targeted therapeutics [11].

Accumulating evidence highlights multiple pathogenic mechanisms underlying diabetic wounds, including persistent inflammation, ferroptosis, oxidative stress, and immune dysregulation. The chronic hyperglycemic microenvironment in diabetic wounds drives sustained inflammatory cell infiltration, perpetuating a state of chronic inflammation [12,13]. This has a similar pathogenesis to DKD. As a chronic refractory wound, diabetic wounds are characterized by a persistent high-glucose microenvironment that drives the accumulation of neutrophils and macrophages, and the continuous release of tumor necrosis factor-α (TNF-α) and interleukin-6 (IL-6). These abnormalities maintain the wound in a chronic inflammatory state, thereby preventing its transition to the proliferative phase [12,13]. Ferroptosis, an iron-dependent, non-apoptotic form of regulated cell death, is characterized by elevated intracellular Fe^2+^ levels, excessive reactive oxygen species (ROS) production, diminished glutathione peroxidase 4 (GPX4) activity, and lipid peroxide accumulation, ultimately leading to cell demise [14,15]. Emerging studies have identified iron metabolism as a key regulator of wound healing [16], and ferroptosis has been documented in diabetic wounds [17,18,19]. The occurrence of ferroptosis impairs the viability of skin fibroblasts and vascular endothelial cells, thereby delaying diabetic wound healing [20,21]. A long-term high-glucose environment in diabetic wounds can also lead to oxidative stress, leading to the depletion of glutathione (GSH), the precursor of GPX4 synthesis, resulting in the accumulation of ROS, which activates lipid peroxidation and causes ferroptosis [22]. In addition, oxidative stress can activate inflammatory pathways such as NF-κB, up-regulate the expression of pro-inflammatory factors, and aggravate local chronic inflammatory response [23]. The vicious cycle of “oxidative stress–inflammation–ferroptosis” seriously inhibits the process of wound repair. Thus, targeting inflammation and ferroptosis represents a promising therapeutic approach for diabetic wound management.

Natural products, renowned for their structural diversity and multi-target bioactivity, serve as invaluable sources for drug discovery. At present, some progress has been made in the research of natural products in the field of diabetic wound healing. Many studies have shown that natural bioactive compounds such as resveratrol and ginsenoside can promote diabetic wound healing by regulating programmed cell death pathways including apoptosis and ferroptosis [24]. Extracts of natural products such as quercetin, curcumin, and paeoniflorin act through antioxidant, antibacterial, anti-inflammatory, and pro-angiogenic mechanisms [25]. Current research on natural products mostly focuses on a single pathway, while the disorder of diabetic wound healing is a complex result of the interaction of multiple pathological factors [26]. Therefore, it is of great significance to clarify the multi-target regulatory mechanism of inflammation and ferroptosis for diabetic wound healing. *Ginkgo biloba* L, a “living fossil” and the sole surviving species of the Ginkgophyta division, is rich in bioactive components such as flavonoids and terpene lactones, exhibiting pharmacological effects including blood circulation promotion and lipid lowering [27]. Clinically, *Ginkgo biloba* L are widely used for cardiovascular and cerebrovascular protection [28]. At present, the pharmacological properties of *Ginkgo biloba* leaves have been extensively studied, but the research on the male flower of *Ginkgo biloba* flower extract (GBF) is still in its infancy [29]. Our previous study showed that the contents of flavonoids (1.42 times higher), amino acids (15.7 times higher) and fatty acids (7.4 times higher) in GBF were significantly higher than those in *Ginkgo biloba* leaf extract. Of the 73 identified flavonoids, 57 were found to be more abundant in GBF [29,30]. These results suggest that GBF may be a superior choice for the source of bioactive compounds compared to the conventionally used *Ginkgo biloba* leaf extract. Notably, GBF showed superior anti-ferroptosis activity in erastin-induced PC12 cells and exerted stronger anti-inflammatory effects through bilobetin and isoginkgetin [29,31]. Therefore, GBF had a stronger inhibitory effect on inflammation and ferroptosis than *Ginkgo biloba* leaf extract. However, the therapeutic potential of GBF in diabetic wound healing has not been fully investigated. Therefore, this research chose to focus on inflammation and ferroptosis to investigate the effect of GBF on diabetic wound healing in the expectation of discovering a novel natural product candidate with potential in diabetic wound treatment.

## 2. Results

### 2.1. Qualitative Analysis of the Total Chemical Composition of GBF

**Figure 1:**
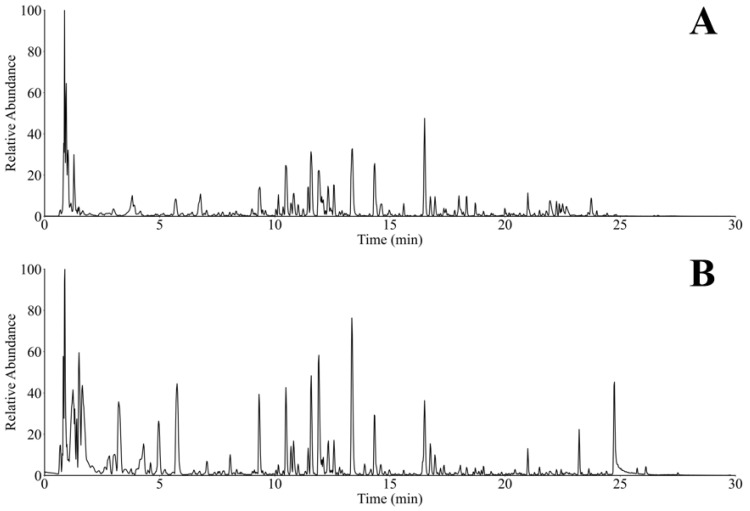
BPI of GBF detected in negative (**A**) and positive (**B**) mode.

The mass spectrometry data collected were processed using the following methods: Progenesis QI 3.0 software (Waters Corp, Mlford, MA, USA) was used. The operation steps were as follows: import raw data, peak extraction and deconvolution processing. The reference material database (TCM Pro 2.0, Beijing Hexin Technology, Beijin, China) and theoretical databases (based on the literature and public databases) were searched. Based on the retention time error of reference materials, mass error of parent ions, matching of secondary fragments, isotope distribution, peak intensity and other factors, the identification results were comprehensively evaluated and analyzed in multiple dimensions, and the final results were obtained. The base peak ion chromatograph (BPI) of GBF is shown in Figure 1. Finally, 123 compounds were identified in GBF (Table 1), including 73 flavonoids, 12 terpenoids, 10 phenolic acids, six alkaloids and five phenylpropanoids. Other chemical classes include anthraquinones, fatty acids, amino acids, purines, and alkaloids.

### 2.2. GBF Promotes Skin Wound Healing in Normal Rats

**Figure 2:**
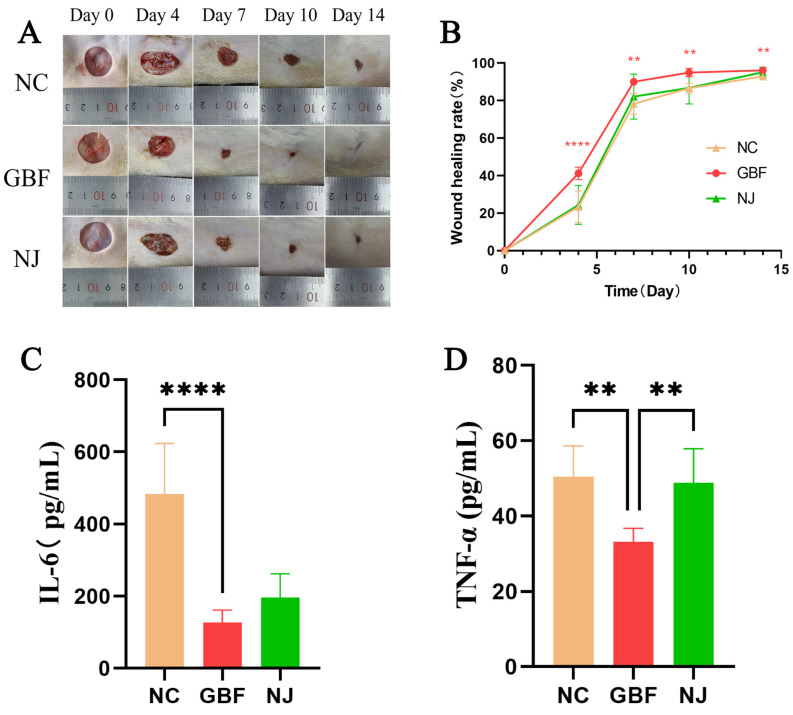
GBF promotes common wound healing in vivo. (**A**) Representative images of wound changes in rats in each group over 14 days. (**B**) Wound healing rate statistics. (**C**,**D**) Content of IL-6 and TNF-α in wound tissue samples on day 14. The results are shown as the mean ± standard deviation (SD), with a sample size of *n* = 11. Statistical significance was determined with the following levels: ** *p* < 0.01, and **** *p* < 0.0001.

To validate whether GBF promotes the healing of normal skin wounds, experiments were conducted in vivo to observe wound area changes over a 14-day period. The wound healing rate increased progressively across all groups over time (Figure 2A,B). On day 4, the recovered wound area rate in the GBF group was extremely significantly higher than that in the control group (*p* < 0.0001), and this superiority persisted until the end of the experiment (*p* < 0.01). Notably, GBF outperformed the NJ (medical biological gel) group, and wounds were nearly fully healed by the 14th day with a 95% healing rate.

Then, the inflammatory cytokines TNF-α and IL-6 in wound tissues were quantified. Compared with the control group (Figure 2C), the GBF group exhibited a dramatic reduction in IL-6 levels (*p* < 0.0001), which is additionally lower than the NJ group. Consistent with IL-6 (Figure 2D), the TNF-α content of the GBF group showed a significant decrease (*p* < 0.01). These findings suggest that GBF exerts potent in vivo anti-inflammatory activity, consistent with our prior research in vitro [31].

### 2.3. GBF Accelerates Skin Wound Healing in Diabetic Rats

**Figure 3:**
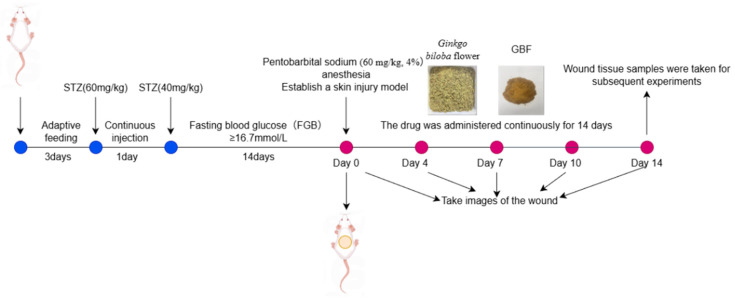
Schematic diagram of the establishment and administration of the skin injury model in diabetic rats.

**Figure 4:**
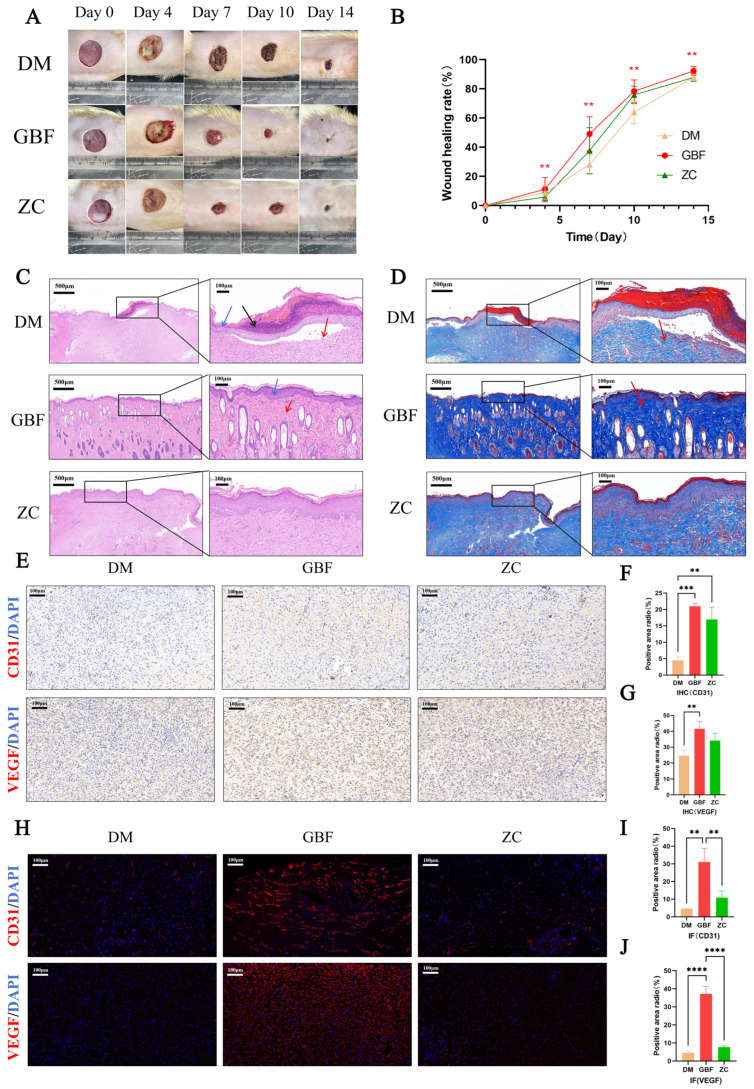
GBF promotes diabetic wound healing in vivo. (**A**) Representative images of wound changes in rats in each group over 14 days. (**B**) Wound healing rate statistics. (**C**) H&E staining was used to evaluate the wound healing status of rats in each group. Epithelial tissue thickness (blue arrow), local necrotic tissue infiltrated by inflammatory cells (black arrow), dermal tissue arrangement (red arrow). Scale bar: 500, 100 μm. (**D**) Masson’s trichrome staining was used to evaluate the wound healing status of rats in each group. Deposition and rearrangement of collagen fibers (red arrows). Scale bar: 500, 100 μm. (**E**–**G**) Immunohistochemistry was used to detect the expression of CD31 and VEGF in rat wound tissue. Scale bar: 100 μm. (**H**–**J**) Immunofluorescence was used to detect the expression of CD31 and VEGF in rat wound tissue. Scale bar: 100 μm. The results are shown as the mean ± standard deviation (SD), with a sample size of *n* = 11. Statistical significance was determined with the following levels: ** *p* < 0.01, *** *p* < 0.001, and **** *p* < 0.0001.

Following confirmation of GBF’s normal wound-repairing efficacy, its therapeutic potential for diabetic wounds was further evaluated. The experimental procedure is shown in Figure 3. As shown in Figure 4A,B, the wound closure area increased gradually in all diabetic rat groups over time. Compared to the DM group, GBF significantly increased the wound healing rate on days 4 (*p* < 0.01), 7 (*p* < 0.01), 10 (*p* < 0.01), and 14 (*p* < 0.01) post-modeling, with the efficacy superior to the positive control drug.

The H&E staining of wound tissues on day 14 (Figure 4C) revealed that wounds in the DM group remained unhealed, characterized by thin epithelial tissue, localized necrotic debris, prominent inflammatory cell infiltration, and loosely arranged dermal tissue. In contrast, the GBF group displayed thicker, fully re-epithelialized wounds, less inflammatory infiltration, denser dermal collagen alignment, and scattered skin appendages. Masson staining of the same regions to assess collagen deposition and remodeling showed that GBF promoted more robust collagen accumulation and fiber rearrangement than that of the DM group (Figure 4D).

Immunohistochemistry (IHC) and immunofluorescence (IF) were used to detect CD31 and VEGF in wound tissues. The IHC results indicated that GBF significantly up-regulated CD31 (*p* < 0.001) and VEGF (*p* < 0.01) expression relative to the DM group (Figure 4E–G). The IF analyses confirmed these findings, with GBF enhancing CD31 (*p* < 0.01) and VEGF (*p* < 0.0001) levels more effectively than the positive drug (Figure 4H–J). Collectively, these data indicated that GBF can facilitate neovascularization in wound tissues.

### 2.4. GBF Suppresses Inflammation, Oxidative Stress and Ferroptosis in Diabetic Wounds

**Figure 5:**
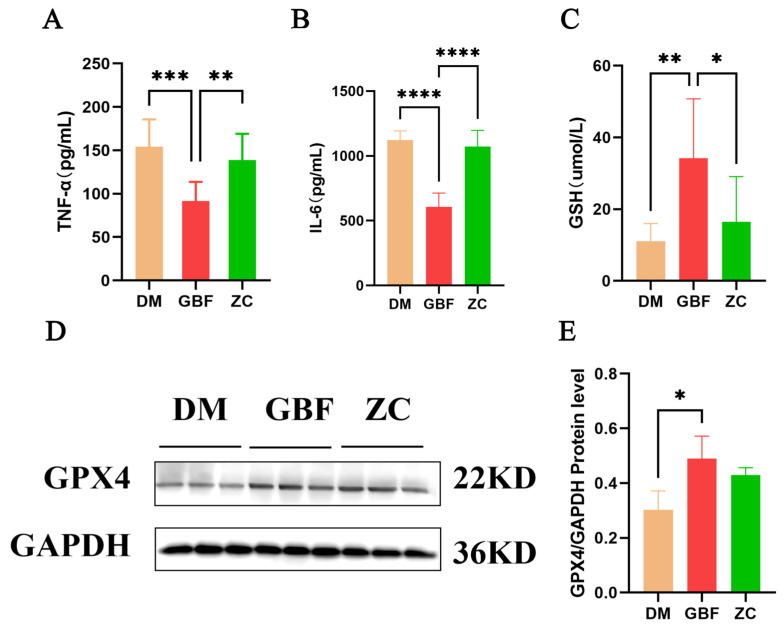
GBF can inhibit inflammation and ferroptosis in diabetic wounds. (**A**–**C**) The content of TNF-α,IL-6 and GSH was quantified using a commercial kit. *n* = 8. (**D**,**E**) The expression level of GPX4 was detected by Western blot. *n* = 3. The results are shown as the mean ± standard deviation (SD). Statistical significance was determined with the following levels: * *p* < 0.05, ** *p* < 0.01, *** *p* < 0.001, and **** *p* < 0.0001.

The inflammatory cytokines TNF-α and IL-6 in diabetic rat wound samples were then measured again. The GBF group showed markedly lower TNF-α (*p* < 0.001) and IL-6 (*p* < 0.0001) levels than the DM group (Figure 5A,B). Additionally, the oxidative stress marker GSH was significantly elevated by GBF compared to the DM group (*p* < 0.0001, Figure 5C). Western blotting was then employed to assess the expression of the ferroptosis marker GPX4 in wound tissues on day 14. The results showed that GBF significantly increased the GPX4 protein levels relative to the DM group (*p* < 0.05) (Figure 5D,E).

### 2.5. Proteomic Analysis in Diabetic Wounds

**Figure 6:**
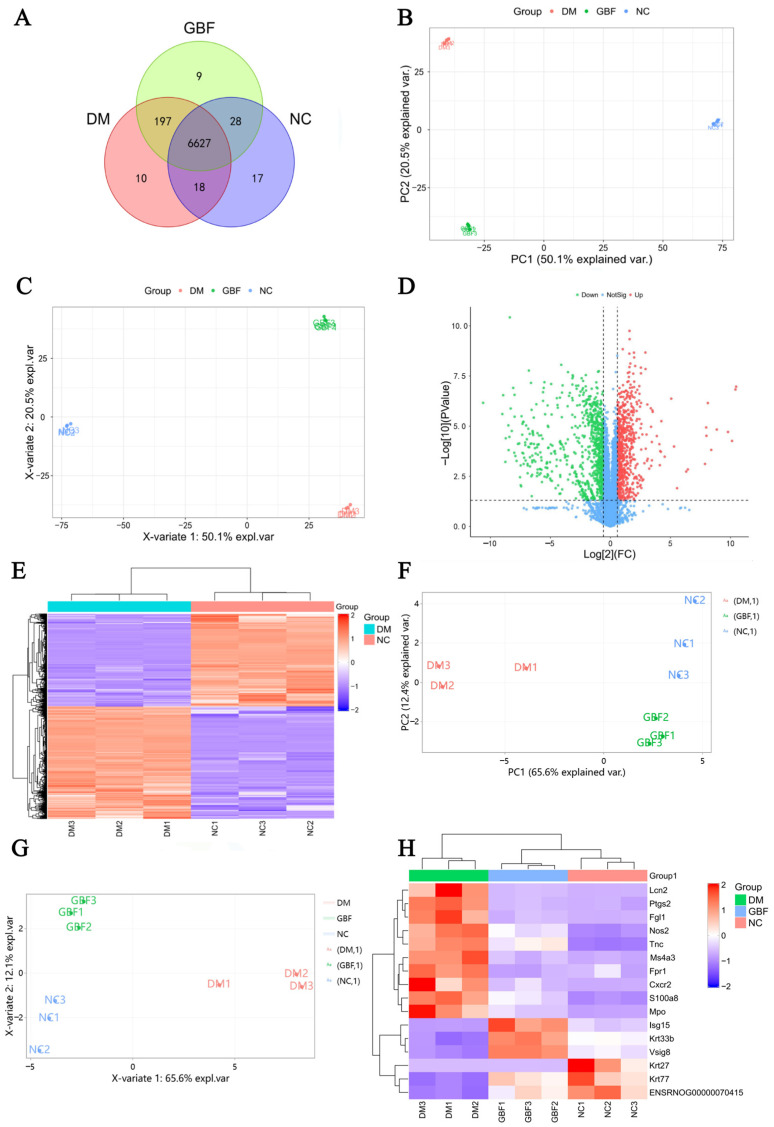

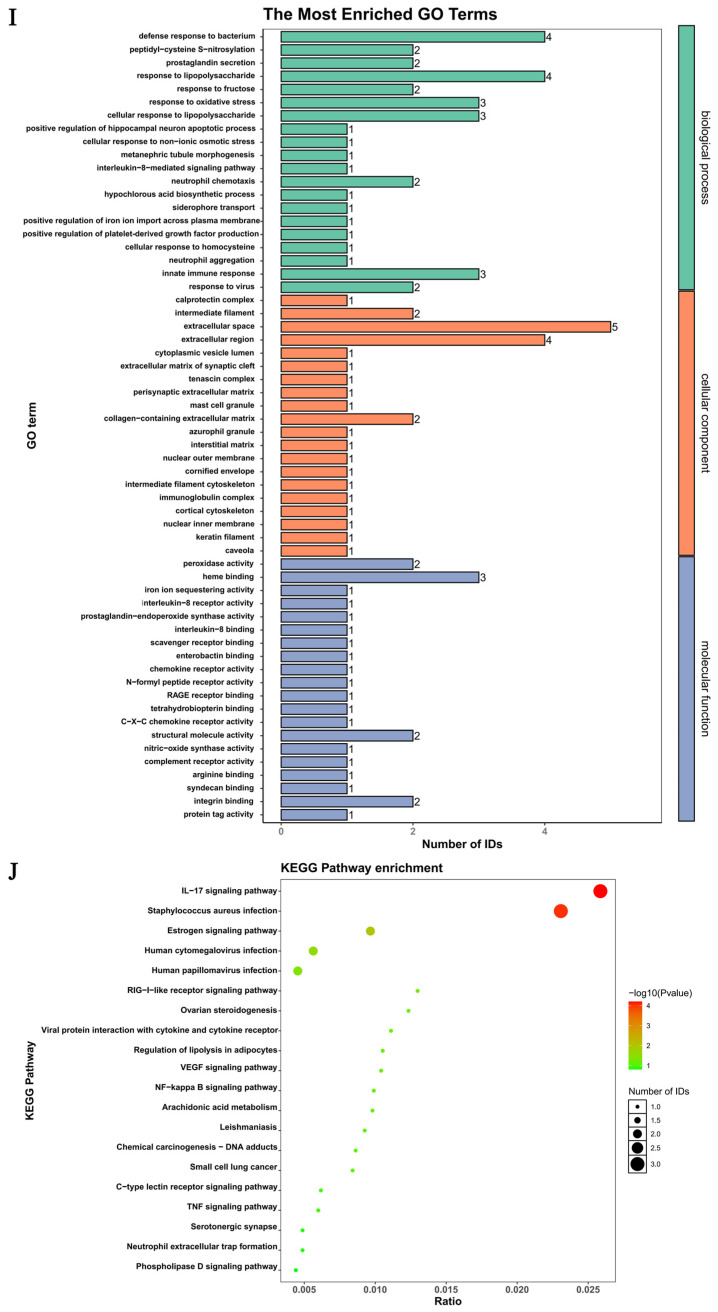
Proteomic analysis revealed the regulation of protein expression in GBF diabetic wounds. (**A**) Venn diagram of proteins between sample groups. (**B**) Plot of principal component analysis (PCA) scores. (**C**) Partial least squares discriminant analysis (PLS-DA) score plot. (**D**) Volcano plot of differentially expressed proteins. Red dots: up-regulated proteins; green dots: down-regulated proteins. (**E**) Heat map of 1578 differentially expressed proteins between NC group and DM group identified by label-free untargeted proteomics. Blue and red indicate down-regulation and up-regulation, respectively. (**F**,**G**) PCA and PLS-DA score map of targeted proteomics analysis. (**H**) Heat map of differentially expressed proteins in NC group, DM group and GBF group shown by targeted proteomics analysis based on proteome relative enrichment matrix (PRM). (**I**) GO enrichment analysis (comparison between DM group and GBF group). (**J**) KEGG pathway enrichment analysis (comparison between the DM group and the GBF group). Pathways were ranked by enrichment ratio. Dot size reflects the amount of protein.

To elucidate the underlying mechanism of GBF in diabetic wound repair, label-free quantitative proteomic analysis was performed on rat skin wound tissue after 14 days of percutaneous wound modeling. Among the 6627 consistently quantified proteins, principal component analysis (PCA) showed significant separation between groups (Figure 6A–C). Based on the criteria of fold change (FC) > 1.5 or < 1/1.5 with a *p* < 0.05, we identified 1578 significantly altered proteins (712 up-regulated and 866 down-regulated) between the NC and DM groups (Figure 6D). Hierarchical clustering analysis revealed that these proteins had unique expression patterns, with color intensity indicating relative abundance (Figure 6E), confirming a significantly altered proteomic profile in the wound tissue of diabetic rats.

Candidate proteins were selected based on two significantly differentially expressed proteins (FC > 1.5 or < 1/1.5, *p* < 0.05) that were associated with ferroptosis and inflammation. Subsequently, PRM validated 27 candidate genes. The results showed that 16 proteins (6 up-regulated and 10 down-regulated) were significantly altered in the DM group. GBF treatment reversed the changes in the above key proteins (Figure 6F–H). These PRM-validated target proteins may be potential regulatory mediators of GBF’s anti-inflammatory and anti-ferroptosis effects in diabetic wounds.

To further characterize the functional annotations of the identified proteins, Gene Ontology (GO) enrichment analysis was performed across three ontological categories: biological process (BP), cellular component (CC), and molecular function (MF) (Figure 6I). The differentially expressed proteins were significantly enriched in a wide range of GO terms, revealing critical insights into the underlying biological mechanisms. Significantly enriched in BP terms associated with inflammatory response were “response to lipopolysaccharide”, “cellular response to lipopolysaccharide” “neutrophil chemotaxis”, and “peptidyl-cysteine S-nitrosylation”. In addition, “response to oxidative stress” was also significantly enriched. It is worth noting that “Multiple terms related to iron metabolism and transport such as” siderophore transport “ and” positive regulation of iron ion import across plasma. “Membrane” was also significantly enriched, suggesting a potential association of ferroptosis in GBF repair of diabetic wounds. In CC classification, enriched terms mainly reflected extracellular matrix and structural protein features, such as “extracellular space”, “extracellular region”, “collagen-containing ECM”, “intermediate filament”, and “cornified envelope”; these results show the remodeling of the extracellular microenvironment and significant changes in the cytoskeleton structure. To demonstrate the relevance of GBF in diabetic skin wound healing or barrier function reconstruction, the MF category revealed enrichment in “heme binding”, “peroxidase activity”, “IL-8 receptor activity “, “chemokine receptor activity”, and “RAGE receptor binding”. These responses are associated with oxidative stress and inflammation. Collectively, the GO enrichment analysis demonstrates that the differentially expressed proteins are predominantly involved in inflammatory and immune responses, oxidative stress regulation, iron homeostasis, and extracellular matrix remodeling.

KEGG pathway enrichment analysis of the proteomic data revealed that GBF may regulate multiple biological processes (Figure 6J). The significantly enriched pathways covered multiple functional domains. Notably, the IL-17 signaling pathway, TNF signaling pathway, and NF-kappa B signaling pathway were identified, suggesting a potential involvement of inflammatory and immune regulatory mechanisms. Additionally, the VEGF signaling pathway was enriched, indicating a possible role in angiogenesis and vascular permeability regulation. Other enriched pathways, such as arachidonic acid metabolism, neutrophil extracellular trap formation, and the phospholipase D signaling pathway, further support the involvement of lipid metabolism and innate immune responses. These enrichment results showed that the differentially expressed proteins were mainly involved in inflammatory signal transduction, immune response and metabolic regulation. The multi-pathway regulation mechanism of GBF provides an important basis for its molecular mechanism of action in diabetic wound repair.

### 2.6. GBF Inhibits Ferroptosis and Enhances Viability in Rat Skin Fibroblasts (RS1)

**Figure 7:**
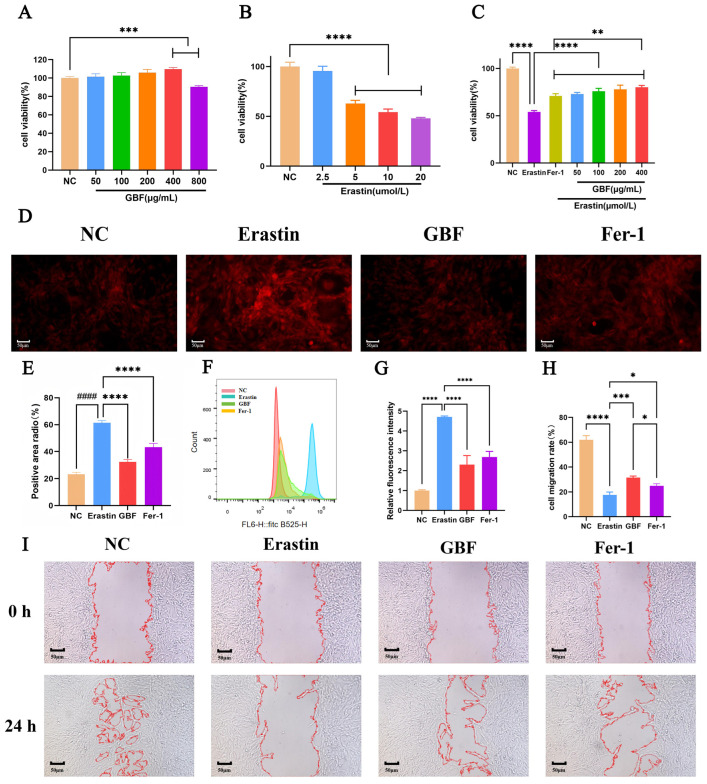
GBF can improve RS1’s activity by inhibiting ferroptosis. (**A**) CCK-8 assays evaluated GBF’s cytotoxicity on RS1 cells. (**B**) CCK-8 assay was used to detect the ferroptosis activity of RS1 cells induced by different concentrations of erastin. (**C**) CCK-8 assay was used to detect the activity of GBF and Fer-1 on ferroptosis cell RS1. (**D**,**E**) The expression intensity of Fe^2+^ in cells was detected by immunofluorescence. Scale bar: 50 μm. (**F**,**G**) The expression of ROS was detected by flow cytometry. (**H**,**I**) Images and data statistics of cell migration area at 24 h in each group. Scale bar: 50 μm. The results are shown as the mean ± standard deviation (SD), with a sample size of *n* = 3. Statistical significance was determined with the following levels: * *p* < 0.05, ** *p* < 0.01, *** *p* < 0.001, **** *p* < 0.0001 and ^####^ *p* < 0.0001.

CCK-8 assays were used to evaluate GBF’s cytotoxicity on RS1 cells. Figure 7A shows that the 50–400 μg/mL GBF promoted RS1 proliferation in a dose-dependent manner. At a concentration of 400 μg/mL, RS1 cell viability was enhanced to approximately 110% relative to the negative control (*p* < 0.001), while the GBF of 800 μg/mL significantly suppressed cell viability (*p* < 0.001). The RS1 cells were then treated with varying concentrations of the ferroptosis inducer erastin. As displayed in Figure 7B, the 5–20 μmol/L erastin remarkably reduced RS1 survival (*p* < 0.0001), and the 10 μmol/L erastin decreased cell viability to nearly 50% (*p* < 0.0001). Thus, the 10 μmol/L erastin was selected to establish an RS1 ferroptosis model for investigating GBF’s inhibitory effects. The 50–400 μg/mL GBF treatment exhibited a dose-dependent rescue of erastin-induced RS1 cells (*p* < 0.0001). At the dose of 400 μg/mL, GBF increased the cell viability to 81% (Figure 7C). Fluorescence microscopy revealed that erastin induction significantly elevated intracellular Fe^2+^ levels (*p* < 0.0001), while 400 μg/mL GBF dramatically reduced Fe^2+^ concentrations (*p* < 0.0001)—with efficacy superior to the positive drug Ferrostatin-1 (Fer-1) (Figure 7D,E). Flow cytometry analysis of reactive oxygen species (ROS) showed that erastin markedly increased the ROS levels in RS1 cells (*p* < 0.0001), whereas GBF significantly attenuated ROS accumulation (*p* < 0.0001) more effectively than Fer-1 (Figure 7F,G), indicating GBF can mitigate oxidative stress in ferroptosis cells. Furthermore, the scratch assays demonstrated that erastin drastically impaired the migration rate of RS1 (*p* < 0.0001) after treatment for 24 h, while GBF significantly restored the migration capacity (*p* < 0.001) with a better efficacy than Fer-1 (Figure 7H,I).

## 3. Materials and Methods

### 3.1. Materials

Fresh male inflorescences of *Ginkgo biloba* L., the sole surviving species of the class Ginkgoopsida within the division Gymnospermae, were manually harvested in April 2023 from Tancheng County, Shandong Province, China. The botanical identity was authenticated by Professor Li of our laboratory. The voucher specimen (Accession No. GBF230415) is deposited in Beijing Academy of Military Medical Sciences.

Medical bio-gels (NJ), mainly composed of sodium alginate and carboxymethyl chitosan, are widely used as wound dressings to accelerate wound healing [91]. Wang et al. found that Zhu Zi ointment (Zi Cao ointment) promoted macrophage polarization by activating the PI3K/AKT pathway, thereby inhibiting inflammation and promoting ulcer wound healing [92]. Therefore, this study selected medical bio-gel as the common wound in vivo and Zi Cao (ZC) ointment as the positive drug for the diabetic wound study.

### 3.2. Determination of Chemical Composition

#### 3.2.1. Sample Preparation

A 50mg sample of GBF and 1 mL of 80% methanol were loaded into a 1.5 mL centrifuge tube and sonicated for 30 min. Then, 0.2 mL of the suspension and 0.2 mL of 80% methanol were transferred to a 1.5 mL tube and centrifuged at 12,000 rpm for 10 min at 4 °C. Finally, 100 μL of the supernatant was injected for testing.

#### 3.2.2. Chromatographic Method

A Vanquish Flex UHPLC chromatograph (Thermo Fisher Scientific, Inc., Waltham, MA, USA) equipped with an ACQUITY UPLC HSS T3 column was used for separation. The mobile phase consisted of water (0.1% formic acid, phase A) and acetonitrile (phase B) at a flow rate of 0.3 mL/min. The column temperature was 40 °C. The mobile-phase gradients were the following: 0–1 min: 2%B; 1–14 min: 2–30% B; 14–25 min: 30–100% B; 25–28 min: 100% B.

#### 3.2.3. Mass Spectrometry Method

The MS data were collected by a hybrid quadrupole orbitrap mass spectrometer (Q Exactive, Thermo Fisher Scientific, Inc., Waltham, MA, USA) equipped with a HESI-II spray probe. The parameters were set as follows: the ion source voltages of positive (ESI+) and negative (ESI-) ions were 3.7 kV and 3.5 kV, respectively. The capillary heating temperature was 320 °C. The sheath gas pressure was 30 psi and the auxiliary gas pressure was 10 psi. The solvent heating evaporation temperature was 300 °C; the sheath gas and auxiliary gas were nitrogen. The collision gas was nitrogen at a pressure of 1.5 mTorr. The mass-to-charge ratio scanning range was from 100 to 1500.

### 3.3. Preparation of Extract

The collected air-dried male inflorescences of *Ginkgo biloba* L were cleaned to remove impurities. Subsequently, 200 g of the material was immersed in 70% aqueous ethanol. After 48 h of maceration, reflux extraction was performed for four cycles, each lasting 2 h. The combined extracts were concentrated under reduced pressure to remove the solvent, yielding a crude extract. The extract was dispersed in a suitable volume of water and then successively extracted with petroleum ether. The extraction was repeated until the petroleum ether layer became colorless. The combined petroleum ether phases were concentrated using a rotary evaporator (BUCHI, Flawil, Switzerland) and then dried under vacuum to obtain the GBF [29,31].

### 3.4. Animals Experiment

Male Sprague Dawley (SD) rats were purchased from Beijing Sibef Biotechnology (Beijing, China) Co., Ltd. The rats were specific-pathogen-free (SPF), aged 6–8 weeks, and weighed 190–210 g. They were housed in a controlled-environment room maintained at 24–26 °C and 40–60% relative humidity, with a 12 h/12 h light–dark cycle. Food and water were available ad libitum. The Experimental Animal Ethics Committee of the Beijing Academy of Military Medical Sciences approved the animal experiments.

### 3.5. Establishment of Skin Injury Model in Normal Rats

The rats were anesthetized by intraperitoneal administration of sodium pentobarbital (60 mg/kg, 4% solution, Beijing, China). The dorsal hair was shaved, and any remaining hair follicles were thoroughly removed using a depilatory cream (Veet, Jingzhou, Hubei, China). Following disinfection with iodophor, a circular full-thickness skin open wound (20 mm in diameter) was marked on the shaved dorsal skin adjacent to the spine using a biopsy punch (Cellvis, Hangzhou, China).

For the wound healing assay, rats were randomly assigned to three groups (*n* = 11): the negative control (NC) group, medical biological gel (NJ) (Zhende Medical, Laoshan,, China) group, and the GBF group. The GBF solution of 20 mg/mL was applied topically to the wounds of each rat in the GBF group by means of spray daily. The NJ and NC groups received an equivalent volume of biological gel applied to cover the wound or normal saline (sprayed), respectively. The wound areas were photographed on days 0, 4, 7, 10, and 14 following the induction of the wounds. The captured images were analyzed using ImageJ software V1.4.4 to quantify the wound area. The wound healing rate was calculated as follows: [(initial wound area—unhealed area)/initial wound area] × 100%. At 14 days, the rats were euthanized, after which the wound tissues were collected for further experiments.

### 3.6. Establishment of Skin Injury Model in Diabetic Rats

Diabetes was induced by two intraperitoneal injections of streptozotocin (STZ, Solarbio, Beijing, China). The first injection was administered at a dose of 60 mg/kg, followed 24 h later by a second injection at 40 mg/kg. Two weeks later, fasting blood glucose (FBG) levels were measured daily for three consecutive days using a glucometer (Yuwell, Danyang, China), with blood samples obtained from the tail vein. Rats with FBG levels ≥ 16.7 mmol/L on all three days were considered diabetic, indicating successful model establishment.

The diabetic skin wound model was established following the procedure described for the normal wound model (Section 2.4). Rats were randomly divided into three groups (*n* = 8): the negative control (NC) group, the Zi Cao ointment (ZC) (Catalong Pharmaceutical, Kunming, China) group, and the GBF group. Treatments were administered, and wound healing rates were assessed as outlined in the normal wound experiment (Section 2.5).

### 3.7. Hematoxylin and Eosin (H&E) Staining

Following the collection of wound skin samples, they were fixed in 4% paraformaldehyde solution (Solarbio, Beijing, China). Subsequently, the samples underwent dehydration using absolute ethanol, were embedded in paraffin, and sectioned into 5 µm thick sections. After H&E (Servicebio, Wuhan, China) staining, the tissue sections were examined under a light microscope (Olympus, Nagano, Japan), and representative images were captured.

### 3.8. Masson’s Trichrome Staining

Wound skin samples were fixed in 4% paraformaldehyde solution (Solarbio, Beijing, China). Subsequently, the samples underwent dehydration using absolute ethanol, were embedded in paraffin, and sectioned into 5 µm thick sections. The sections were then subjected to Masson’s trichrome (Servicebio, Wuhan, China) staining. Finally, the stained sections were examined and imaged using a light microscope (Olympus, Nagano, Japan).

### 3.9. Immunohistochemistry and Immunofluorescence

The 4% paraformaldehyde (Solarbio, Beijing, China) fixed tissue sections were subjected to dewaxing and dehydration procedures, followed by antigen retrieval and slide mounting. Endogenous peroxidase activity was then quenched, and non-specific binding sites were blocked with serum. The sections were subsequently incubated overnight at 4 °C with the following primary antibodies: rabbit anti-CD31 antibody (1:500, Servicebio, Wuhan, China) and rabbit anti-VEGF antibody (1:250, Servicebio, Wuhan, China). After washing with phosphate-buffered saline (PBS), the sections were incubated with HRP-conjugated goat anti-rabbit IgG secondary antibody (Servicebio, Wuhan, China) at room temperature for 50 min. Counterstaining was performed using hematoxylin, and sample images were acquired using a light microscope (Olympus, Nagano, Japan). Finally, the quantitative image analysis was conducted using Image J software V1.4.4.

The subsequent sample preparation and antigen retrieval steps remain unchanged as outlined in the Immunohistochemistry section. The sections were subsequently incubated overnight at 4 °C with the following primary antibodies: rabbit anti-CD31 antibody (1:100, Servicebio, Wuhan, China) and rabbit anti-VEGF antibody (1:200, Servicebio, Wuhan, China). After washing with phosphate-buffered saline (PBS), the sections were incubated with CY3-conjugated goat anti-rabbit IgG secondary antibody (Servicebio, Wuhan, China) at room temperature for 50 min. The nuclei were counterstained with DAPI (Servicebio, Wuhan, China), and tissue autofluorescence was quenched using an autofluorescence quenching agent (Servicebio, Wuhan, China). Finally, the sections were imaged under a fluorescence microscope (Nikon, Kanagawa, Japan) and the images were analyzed using ImageJ software V1.4.4.

### 3.10. Determination of GSH, TNF-α, IL-6, and GPX4

GSH levels in wound tissue samples were determined using a Total Glutathione/Oxidized Glutathione (T-GSH/GSSG) Assay Kit (Jiancheng Technology, Nanajing, China), strictly following the manufacturer’s instructions.

TNF-α and IL-6 levels in wound tissue samples were determined using a Rat TNF-α Precoated ELISA Kit (Dayou Biology, Shenzhen, China) and Rat IL-6 Precoated ELISA Kit (Dayou Biology, Shenzhen, China), strictly following the manufacturer’s instructions.

The tissues were homogenized in RIPA lysis buffer (Applygen, Beijing, China) to extract the total protein. Protein concentrations were determined using a BCA assay (Applygen, Beijing, China) to ensure equal loading across all wells. The proteins were then separated by electrophoresis on 12.5% SDS–polyacrylamide gels (Applygen, Beijing, China) and transferred onto polyvinylidene difluoride (PVDF) membranes (Merck, Shanghai, China). After blocking, the membranes were incubated overnight at 4 °C with the following primary antibodies: anti-GPX4 (1:1000, Abcam, Cambridge, UK) and anti-GAPDH (1:500, Servicebio, Wuhan, China). Subsequently, the membranes were incubated with horseradish peroxidase (HRP)-conjugated secondary antibodies (Abcam, Cambridge, UK) for 1 h at room temperature. The protein bands were visualized using a chemiluminescence imaging system (Bio-Rad Laboratories, Hercules, CA, USA), and their relative intensities were quantified with ImageJ software V1.4.4.

### 3.11. Proteomics Analysis

Data-Independent Acquisition Proteomics was used to compare the differences in protein expression among the NC, DM and GBF groups. In addition, a parallel response monitoring (PRM) technique was used to verify the regulatory effect of GBF on the differential protein expression in the wounds of diabetic rats. Protein identification was performed by UPLC-MS/MS (Qinglian Biotechnology, Beijing, China). Data processing was done using ProteomeDiscoverer (v2.4) and Skyline software (v23.1).

### 3.12. Cell Culture

RS1 cells (Fuheng Biology, Shanghai, China) were cultured in DMEM medium (Macgene Biotechnology, Beijing, China) containing 1% streptomycin and penicillin (Macgene Biotechnology, Beijing, China), and 10% fetal bovine serum (FBS) (Gibco, Grand Island, NY, USA) in a cell incubator (Thermo Fisher Scientific, Waltham, MA, USA, 37 °C, 5% CO_2_).

### 3.13. Cell Viability

The 5 × 10^3^ cells were seeded into 96-well plates in a cell culture incubator for 8 h. Subsequently, GBF solutions at varying concentrations (0, 50, 100, 200, 400, 800 μg/mL) were added to evaluate the cytotoxicity of GBF. Under identical culture conditions, erastin (Selleck, Houston, TX, USA) at concentrations of 0, 2.5, 5, 10, and 20 μmol/L was administered to determine the optimal concentration for inducing ferroptosis. Finally, GBF solutions at different concentrations (50, 100, 200, 400 μg/mL) were added, with 1 μmol/L ferroptosis inhibitor Ferrostatin-1 (Fer-1, Selleck, Houston, TX, USA) serving as a positive control, to investigate the inhibitory effect of GBF on ferroptosis in RS1 cells. Twenty-four hours after treatment initiation, 10 μL of CCK-8 reagent (Meilun Biology, Dalian, China) was added to each well, followed by incubation in a 37 °C incubator for 40 min. Absorbance was measured at a wavelength of 450 nm using a microplate reader (Thermo Sciectific, Singapore) and cell viability was subsequently calculated and compared.

### 3.14. ROS and Fe^2+^ Determination

The DCFH-DA (Elabscience, Wuhan, China) was used to detect ROS levels. The experimental method was to inoculate 5 × 10^5^ cells into 6-well plates and divide them into the following groups, NC (negative control), erastin (10 μmol/L), Fer-1 (1 μmol/L), and GBF (400 μg/mL), and they were then cultured in a cell incubator for 8 h. The cells were washed twice with PBS and then incubated with DCFH-DA at 37 °C for 30 min in the dark. Following a final wash, cells were collected and analyzed by flow cytometry to determine and compare intracellular ROS levels across groups.

The 575 Red Fe^2+^ Dye (BioTracker, St. Louis, MO, USA) was used to detect Fe^2+^ levels. The experimental method was to inoculate 5 × 10^5^ cells into 6-well plates and divide them into the following groups, NC, erastin (10 μmol/L), Fer-1 (1 μmol/L) and GBF (400 μg/mL), and they were then cultured in a cell incubator for 8 h. The cells were washed twice with serum-free medium and then incubated with 575 Red Fe^2+^ Dye at 37 °C for 60 min in the dark. Following a final wash, cell fluorescence intensity was measured using fluorescence microscopy.

### 3.15. Scratch Assay

To investigate the effect of GBF on RS1 cell migration, a scratch assay was conducted on a confluent cell monolayer. The specific steps were to inoculate cells into 6-well plates and divide them into the following groups: NC, erastin (10μmol/L), Fer-1 (1 μmol/L), and GBF (400 μg/mL). Once the cell density reached 95%, the cells were scratched using a 200 μL sterile plastic tip. Following this, the culture was sustained using a serum-free medium. The cell migration of each group was observed by a light microscope (Olympus, Nagano, Japan) after 24 h.

### 3.16. Statistical Analysis

The data were expressed as the mean value ± standard deviation. A one-way analysis of variance was conducted utilizing the GraphPad Prism, version 9.0.0 (GraphPad Software, San Diego, CA, USA), followed by the Dunnett multiple comparison test. All data were analyzed by pairwise comparison among multiple groups. Statistically speaking, p values are labeled as * *p* < 0.05, ** *p* < 0.01, *** *p* < 0.001, **** *p* < 0.0001.

## 4. Discussion

Diabetic wounds are one of the most serious complications of diabetes, with a large number of patients and a high disability and mortality rate. The adverse impact of diabetic ulcers not only seriously affects the quality of life of patients but also brings a heavy economic burden to society [93]. The process of wound healing mainly consists of four overlapping and distinct stages: hemostasis, inflammation, proliferation, and remodeling. These stages involve cell migration and proliferation, extracellular matrix deposition, and tissue remodeling [94]. Numerous studies have shown that various factors, such as persistent inflammation, impaired angiogenesis, oxidative stress, and slow cell growth in diabetic wounds, can have detrimental effects on wound healing [95,96,97,98]. Some research has also reported that ferroptosis inhibits wound healing in diabetic wounds [20,21,99].

Natural traditional Chinese medicine is widely used in the treatment of diabetic wounds. Total iridoid glycoside extract of L. rotata can accelerate diabetic wound healing through NRF2/COX2 axis [100]. Lonicerin facilitates angiogenesis and diabetic wound healing via Sirt1-mediated autophagy activation [101]. *Ginkgo biloba* L is a pharmacologically rich natural traditional Chinese medicine with documented antioxidant, anti-radiation, and cardioprotective properties [102]. Our previous studies have shown that GBF can inhibit ferroptosis in human umbilical vein endothelial cells by down-regulating ACSL4 expression and up-regulating GPX4 expression [31], and also inhibit ferroptosis in nerve cells by inhibiting ROS, LPO destruction and Fe^2+^ elevation [29]. GBF-8, a subcomponent isolated from GBF, reduced neuroinflammation and identified the Slc7a11-Eif4ebp1 axis as the main target of GBF-8 in inhibiting ferroptosis [103]. This is due to its potential active ingredient. The flavonoid ginkgetin can significantly reduce the expression of TNF-α, IL-6, IL-1β and other pro-inflammatory factors by inhibiting the TLR4/NF-κB signaling pathway, while up-regulating the anti-inflammatory factor IL-10 [104]. The flavonoid quercetin can inhibit ferroptosis of endothelial cells by regulating the KEAP1/NRF2/GPX4 signaling pathway [105]. Ginkgolide B, a terpene lactone, can alleviate oxidative stress and ferroptosis by inhibiting GPX4 ubiquitination [106]. We extended this investigation to examine its effects on diabetic wound healing, focusing on inflammation and ferroptosis regulation in vivo. TNF-α and IL-6 are pivotal pro-inflammatory cytokines. Their overexpression in wound tissue is a major barrier to healing [107,108]. In a normal rat wound model, GBF accelerated wound closure and reduced TNF-α/IL-6 levels, confirming its pro-healing and anti-inflammatory effects. GSH is a potent biological antioxidant that mitigates oxidative stress and plays an important role in the ferroptosis pathway [109,110]. The increase in the GSH content in the wound can promote wound healing [111]. In the diabetic rat model induced by multiple STZ injections, the wound tissue levels of TNF-α, IL-6, and GSH were measured. Compared with the DM group, GBF treatment significantly reduced the levels of the pro-inflammatory cytokines TNF-α and IL-6, while increasing the content of the antioxidant GSH. These results suggest that GBF promotes diabetic wound healing by mitigating inflammation and alleviating oxidative stress. GPX4 plays a pivotal role in clearing excess lipid peroxides and is a central regulator of ferroptosis [112]. It mitigates ferroptosis induced by peroxide accumulation by catalyzing the GSH-dependent reduction of lipid hydroperoxides [113]. Western blot analysis demonstrated that GBF treatment up-regulated GPX4 expression compared to the DM group, confirming that GBF can inhibit ferroptosis in diabetic wounds.

VEGF is a key signaling molecule that specifically initiates angiogenesis, promoting the sprouting and growth of new blood vessels [114]. In contrast, CD31 is a transmembrane protein highly expressed at endothelial cell junctions. It serves as a marker of mature vascular endothelium, primarily involved in intercellular adhesion and the maintenance of vascular integrity and permeability, indicating the stabilization of nascent vessels into functional structures [115]. The concurrent up-regulation of both markers in wound tissue is a recognized hallmark of active and maturing angiogenesis. Our IHC and IF analyses of diabetic wound samples revealed that GBF treatment significantly increased the expression levels of both VEGF and CD31 compared to the DM group. This finding indicates that GBF effectively promotes neovascularization in diabetic wounds. Furthermore, histological assessment via H&E and Masson staining demonstrated that the GBF group exhibited superior wound coverage, along with enhanced collagen fiber deposition and remodeling relative to the DM group. Collectively, these results demonstrate that GBF facilitates diabetic wound healing by stimulating functional angiogenesis and improving the quality of tissue repair.

Proteomic analysis revealed that GBF modulates multiple pathways associated with inflammation, oxidative stress, ferroptosis, and angiogenesis in diabetic wounds. Label-free quantitative proteomics identified 1578 differentially expressed proteins between the NC and DM groups (FC > 1.5 or < 1/1.5, *p* < 0.05). Parallel reaction monitoring (PRM) validated 16 proteins (6 up-regulated, 10 down-regulated) in the DM group, whose abnormal expression was reversed by GBF treatment. GO enrichment analysis showed that the differentially expressed proteins were predominantly involved in inflammatory responses (response to lipopolysaccharide, neutrophil chemotaxis), oxidative stress, iron metabolism (siderophore transport, positive regulation of iron ion import), and extracellular matrix remodeling. KEGG pathway enrichment analysis identified key pathways including IL-17, TNF, NF-κB, and VEGF signaling, as well as arachidonic acid metabolism and neutrophil extracellular trap formation. Collectively, these proteomic findings indicate that GBF promotes diabetic wound healing through multi-pathway regulation of inflammation, oxidative stress, ferroptosis, and angiogenesis.

Dermal fibroblasts are pivotal functional cells in skin wound repair, driving healing through proliferation, redifferentiation, and the secretion of extracellular matrix components such as collagen and fibronectin across all four phases of wound healing. Insufficient fibroblast presence in wound skin is a key factor contributing to impaired healing in chronic conditions like diabetic wounds [116]. The occurrence of ferroptosis in fibroblasts is one of the factors affecting diabetic wound recovery [112]. In our experiments in vitro, various concentrations of GBF significantly restored the viability and migration rate of RS1 dermal fibroblasts that had been compromised by the ferroptosis inducer erastin. This indicates that GBF can promote fibroblast growth and function, thereby supporting its promoting effects on diabetic wound closure. Ferroptosis is characterized by Fe^2+^ overload and excessive generation of reactive ROS [14,117]. In erastin-induced ferroptotic RS1 cells, we further observed that GBF treatment effectively reduced both the Fe^2+^ and ROS levels. Taken together, these findings suggest that GBF inhibits ferroptosis in dermal fibroblasts, which may in turn facilitate angiogenesis and enhance the healing of diabetic wounds.

Although GBF has been shown to regulate key ferroptosis-related proteins (GPX4) and inflammatory cytokines (TNF-α, IL-6), the upstream signaling pathways mediating these effects, such as the involvement of the Nrf2 and NF-κB signaling pathways, have not been systematically investigated. In the future, further studies using pathway-specific inhibitors or gene-silencing technology are needed to elucidate the precise molecular mechanism. Complex extract components may create potential confounding factors. GBF is a multicomponent extract containing 123 identified compounds, including flavonoids, terpenoids and other substances. Although this complexity may enhance their multi-target therapeutic utility, it also poses challenges for quality control, batch-to-batch consistency, and regulatory approval. We plan to establish a component separation platform to purify flavonoid and terpene lactone monomers individually, and to conduct comparative experiments to further verify their independent biological functions. Furthermore, based on the structural information of the chemical components, we intend to systematically delineate the structure–activity relationships of these compounds, clarify the impact of different core skeletons and functional groups on their pharmacological activities, and elucidate the synergistic mechanisms by which these components collectively improve diabetic wound healing.

## 5. Conclusions

This study demonstrates that GBF promotes diabetic wound healing through two aspects. On one hand, GBF inhibits key pathological processes—namely ferroptosis, inflammation, and oxidative stress. On the other hand, it enhances repair mechanisms by stimulating angiogenesis and tissue remodeling. Furthermore, GBF was found to promote the proliferation and migration of dermal fibroblasts, an effect closely associated with its inhibition of ferroptosis in these cells. Collectively, these findings identify GBF as a novel and promising natural product candidate for the development of therapeutics aimed at treating diabetic wounds.

## Figures and Tables

**Table 1:** Analysis and identification of chemical constituents in GBF.

No.	tR/ (min)	Name	Formula	*m*/*z*	Expected *m*/*z*	Adducts	Reference
1	0.91	Stachydrine	C_7_H_13_NO_2_	144.1019	144.1019	M + H	[32]
2	1.27	Nicotinamide	C_6_H_6_N_2_O	123.0557	123.0557	M + H	
3	1.50	Dl-isoleucine	C_6_H_13_NO_2_	132.1020	132.1019	M + H	[33]
4	1.61	α-arbutin	C_12_H_16_O_7_	317.0879	317.0878	M-H, M + FA-H	[34]
5	2.34	Hordenine	C_10_H_15_NO	166.1226	166.1227	M + H	[35]
6	2.53	4-hydroxybenzoic acid	C_7_H_6_O_3_	156.0654	156.0654	M + NH4	[36]
7	2.68	4-methoxypyridoxine	C_9_H_13_NO_3_	184.0968	184.0968	M + H	[37]
8	3.14	3-(4-hydroxy-3-methoxyphenyl) propane-1,2-diol	C_10_H_14_O_4_	181.0858	181.0858	M + H-H_2_O	[36]
9	3.40	Cardiospermin	C_11_H_17_NO_7_	274.0935	274.0932	M-H	[38]
10	4.02	Dl-pantothenic acid	C_9_H_17_NO_5_	220.1177	220.1180	M + H-H_2_O, M + H, M + Na	[39]
11	4.27	6-hydroxykynurenate	C_10_H_7_NO_4_	206.0446	206.0448	M + H	[40]
12	4.65	Geniposidic acid	C_16_H_22_O_10_	373.1144	373.1140	M-H	[41]
13	5.07	Neochlorogenic acid	C_16_H_18_O_9_	353.0883	353.0878	M-H	[36]
14	5.12	(_)-epigallocatechin-(4beta-> 8)-(_)-epicatechin	C_30_H_26_O_13_	593.1311	593.1300	M-H	[42]
15	5.46	3,4-dihydroxybenzaldehyde	C_7_H_6_O_3_	139.0389	139.0390	M + H	[43]
16	5.67	Esculin	C_15_H_16_O_9_	339.0723	339.0721	M-H	[36]
17	6.00	Bergenin	C_14_H_16_O_9_	327.0725	327.0721	M-H	[44]
18	6.16	Hypaphorine	C_14_H_18_N_2_O_2_	247.1437	247.1441	M + H	[45]
19	6.36	Quercetin_3-o-rhamnoside_7-o-(6-feruloylglucosyl-(1–3)-rhamnoside)	C_33_H_40_O_22_	787.1951	787.1938	M-H	
20	6.44	Isorhamnetin_3-arabinoglucoside	C_27_H_30_O_16_	611.1599	611.1607	M + H	[43]
21	6.98	Esculetin	C_9_H_6_O_4_	179.0337	179.0339	M + H	[36]
22	7.06	3,4,4a,5,6,7-hexahydro_1,1,4a-trimethyl-2(1 h)-naphthalenone	C_13_H_20_O	193.1585	193.1587	M + H	[46]
23	7.30	Quercetin-3-o-beta-d-glucosyl-(1–2)-alpha-l-rhamnoside	C_27_H_30_O_17_	625.1417	625.1410	M-H	[37]
24	7.79	Aromadendrin	C_15_H_12_O_6_	289.0700	289.0707	M + H-H_2_O, M + H	[47]
25	7.96	(_)-epicatechin	C_15_H_14_O_6_	335.0777	335.0772	M-H, M + FA-H	[48]
26	8.08	Sinapine thiocyanate	C_16_H_24_NO_5_+	310.1645	310.1649	M + H	[49]
27	8.22	Isovanillic acid	C_8_H_8_O_4_	169.0493	169.0496	M + H	[50]
28	8.45	Kaempferol_3-o-glucosy l (1–2) rhamnoside	C_27_H_30_O_15_	593.1518	593.1512	M-H	[51]
29	9.34	Neoisorutin	C_27_H_30_O_16_	611.1598	611.1607	M + H, M + Na, 2 M + H	[52]
30	9.44	Isorhamnetin_3-o-rutinoside	C_28_H_32_O_16_	623.1622	623.1617	M-H	[36]
31	9.46	Pinoresinol diglucoside	C_32_H_42_O_16_	727.2467	727.2455	M-H, M + FA-H	[36]
32	9.47	Myricetin	C_15_H_10_O_8_	319.0442	319.0449	M + H	[53]
33	9.62	Isorhamnetin3-o-beta-D-glucopyranosyl-(1–2)-alpha-l-rhamnopyranoside	C_28_H_32_O_16_	625.1754	625.1763	M + H-H_2_O, M + H	[36]
34	9.96	Eucarvone	C_10_H_14_O	151.1116	151.1118	M + H	[50]
35	10.19	Secoisolariciresinol diglucoside	C_32_H_46_O_16_	731.2788	731.2768	M-H, M + FA-H	[54]
36	10.35	Typhaneoside	C_34_H_42_O_20_	769.2207	769.2197	M-H, M + FA-H	[51]
37	10.37	Isorhamnetin-3-O-rutinoside	C_28_H_32_O_16_	625.1754	625.1753	M+H	
38	10.41	Bilobalide	C_15_H_18_O_8_	325.0928	325.0929	M-H	[36]
39	10.48	Rutin	C_27_H_30_O_16_	609.1464	609.1461	M-H	[36]
40	10.55	Quercetin_3-o-rhamnopyranoside	C_21_H_20_O_11_	447.0934	447.0933	M-H	[36]
41	10.55	Quercetin 3-o-alpha-l-[6í-p-coumaroyl-beta-D-Glucopyranosyl-(1-> 2)- rhamnopyranoside]-7-o-beta-d-glucopyranoside	C_42_H_46_O_23_	917.2373	917.2357	M-H	[36]
42	10.69	Kaempferol 3-o-alpha-l-rhamnopyranosyl(1-> 6)-beta-d-galactopyranoside	C_27_H_30_O_15_	595.1647	595.1658	M + H, M + Na, 2 M + H	[36]
43	10.70	Kaempferol 3-o-rhamnoside	C_21_H_20_O_10_	431.0982	431.0984	M-H	[54]
44	10.74	Kaempferol 3-o-alpha-l-[6í-p-coumaroyl-beta-D-Glucopyranosyl-(1-> 2)-rhamnopyranoside]-7-o-beta-d-glucopyranoside	C_42_H_46_O_22_	901.2424	901.2408	M-H	[36]
45	10.82	Hyperoside	C_21_H_20_O_12_	465.1020	465.1028	M + H, M + Na	[55]
46	10.96	Ginkgolid j	C_20_H_24_O_10_	469.1351	469.1352	M-H, M + FA-H	[56]
47	11.01	Laricitrin	C_16_H_12_O_8_	333.0596	333.0605	M + H	[57]
48	11.23	Ginkgolide c	C_20_H_24_O_11_	439.1247	439.1246	M-H, M + FA-H	[36]
49	11.25	Gibberellic acid	C_19_H_22_O_6_	347.1481	347.1489	M + H-H_2_O, M + H	[41]
50	11.29	Isoquercitrin	C_21_H_20_O_12_	463.0887	463.0882	M-H	[36]
51	11.45	Tricetin	C_15_H_10_O_7_	303.0492	303.0500	M + H	[58]
52	11.58	Cynaroside	C_21_H_20_O_11_	449.1069	449.1079	M + H	[59]
53	11.58	Nicotiflorin	C_27_H_30_O_15_	593.1512	593.1512	M-H	[36]
54	11.69	Cosmetin	C_21_H_20_O_10_	431.0985	431.0984	M-H	[36]
55	11.71	Perlolyrine	C_16_H_12_N_2_O_2_	265.0966	265.0972	M + H	[57]
56	11.88	(e)-4-(4-hydroxy-2,6,6-trimethylcyclohexen_1-yl) but-3-en-2-one	C_13_H_20_O_2_	209.1531	209.1536	M + H	
57	11.92	Isorhamnetin-3-o-neohespeidoside	C_28_H_32_O_16_	625.1749	625.1763	M + H, M + Na, 2 M + H	[36]
58	11.92	Tectoridin	C_22_H_22_O_11_	463.1224	463.1235	M + H	[60]
59	11.94	Astragalin	C_21_H_20_O_11_	449.1069	449.1079	M + H, M + Na	[36]
60	12.09	Syringetin_3-rutinoside	C_29_H_34_O_17_	653.1730	653.1723	M-H, M + FA-H	
61	12.29	Sophoricoside	C_21_H_20_O_10_	433.1121	433.1130	M + H, 2 M + H	[61]
62	12.31	Isorhamnetin 3-galactoside	C_22_H_22_O_12_	477.1038	477.1038	M-H	[62]
63	12.48	Dihydroalangionoside A	C_19_H_36_O_8_	392.2402	375.2369	M + H-H_2_O, M +	[63]
64	12.53	Kaempferol 3-arabofuranoside	C_20_H_18_O_10_	417.0830	417.0827	M-H	[61]
65	12.60	Hesperidin	C_28_H_34_O_15_	609.1830	609.1825	M-H	[64]
66	12.81	Thermopsoside	C_22_H_22_O_11_	463.1226	463.1235	M + H, 2 M + H	[65]
67	12.89	Trifolin	C_21_H_20_O_11_	449.1070	449.1079	M + H	[66]
68	13.09	Rhoifolin	C_27_H_30_O_14_	577.1571	577.1563	M-H	[67]
69	13.34	Quercetin 3-o-alpha-l-[6í-p-coumaroyl-beta-D-glucopyranosyl-(1-> 2)-rhamnopyranoside]	C_36_H_36_O_18_	755.1834	755.1829	M-H	[36]
70	13.65	Coniferin	C_16_H_22_O_8_	387.1302	387.1302	M + FA-H	[68]
71	13.76	Bilobanone	C_15_H_20_O_2_	215.1427	215.1430	M + H-2H_2_O	[69]
72	14.17	Daidzein	C_15_H_10_O_4_	255.0644	255.0652	M + H	[61]
73	14.32	Kaempferol 3-o-alpha-l-[6í-p-coumaroyl-beta-D-glucopyranosyl-(1-> 2)-rhamnopyranoside]	C_36_H_36_O_17_	739.1887	739.1880	M-H	[36]
74	14.41	Isorhamnetin 3-o-a-l-[6,-p-coumaroyl-b-D-glucopyranosyl-(1,2)-rhamnopyranoside]	C_37_H_38_O_18_	769.1993	769.1985	M-H, M + FA-H	[61]
75	14.43	Alangionoside A	C_19_H_34_O_8_	373.2213	373.2213	M + H-H_2_O	[70]
76	14.59	Ginkgolid a	C_20_H_24_O_9_	409.1484	409.1493	M + H-2H_2_O	[36]
77	14.63	Ginkgolide b	C_20_H_24_O_10_	423.1299	423.1297	M-H, M + FA-H	[36]
78	14.8	Dihydrodendranthemoside A	C_19_H_36_O_8_	415.2321	415.2321	M + Na	[71]
79	15.01	Luteolin	C_15_H_10_O_6_	285.0405	285.0404	M-H	[36]
80	15.28	Andrographolide	C_20_H_30_O_5_	349.2023	349.2020	M-H	[72]
81	15.42	Tiliroside	C_30_H_26_O_13_	593.1310	593.1300	M-H	[73]
82	15.67	Calycosin	C_16_H_12_O_5_	283.0612	283.0612	M-H	[74]
83	16.32	Trans-caffeic acid	C_9_H_8_O_4_	163.0386	163.0386	M + H-H_2_O	[61]
84	16.50	Naringenin	C_15_H_12_O_5_	271.0611	271.0612	M-H	[75]
85	16.72	Silibinin	C_25_H_22_O_10_	481.1142	481.1140	M-H	[76]
86	16.76	Genistein	C_15_H_10_O_5_	271.0593	271.0601	M + H	[74]
87	16.93	Hesperetin	C_16_H_14_O_6_	301.0715	301.0717	M-H	[70]
88	16.94	4,4,-dihydroxy-3,3,-imino-di-benzoic acid	C_14_H_11_NO_6_	289.0594	289.0594	M + H	[60]
89	16.97	Kaempferol	C_15_H_10_O_6_	285.0403	285.0404	M-H	[36]
90	17.20	Diosmetin	C_16_H_12_O_6_	301.0699	301.0707	M + H	[77]
91	17.36	Isorhamnetin	C_16_H_12_O_7_	317.0648	317.0648	M + H	[36]
92	17.53	(1ar,4as,7r,7ar,7br)-1,1,7-trimethyl_4-methylidenedecahydro-1 h-cyclopropa(e)azulen_7-ol	C_15_H_24_O	221.1895	221.1900	M + H	[61]
93	17.71	Syringetin	C_17_H_14_O_8_	347.0754	347.0762	M + H	[70]
94	17.75	Tricin	C_17_H_14_O_7_	331.0803	331.0813	M + H	[74]
95	18.05	Amentoflavone	C_30_H_18_O_10_	537.0827	537.0827	M-H	[36]
96	18.34	Chrysoeriol	C_16_H_12_O_6_	301.0698	301.0707	M + H	[71]
97	18.77	Ginkgetin 7-o-beta-d-glucopyranoside	C_38_H_32_O_15_	727.1684	727.1668	M-H, M + FA-H	[78]
98	18.93	Isoginkgetin_7-o-beta-d-glucopyranoside	C_38_H_32_O_15_	729.1795	729.1814	M + H	[78]
99	18.93	3-[5,7-dihydroxy_2-(4-methoxy-phenyl)-4-oxo-4 h-chromen_8-yl]-4-methoxy benzoic acid	C_24_H_18_O_8_	435.1065	435.1075	M + H	
100	19.02	Hexadecanoic acid	C_16_H_32_O_2_	301.2385	301.2385	M + FA-H	[79]
101	19.04	5,-methoxybilobetin	C_32_H_22_O_11_	583.1221	583.1235	M + H	
102	19.08	Parthenolide	C_15_H_20_O_3_	249.1478	249.1485	M + H	[80]
103	19.15	Chrysin	C_15_H_10_O_4_	255.0644	255.0652	M + H	[81]
104	19.24	Roburic acid	C_30_H_48_O_2_	441.3715	441.3727	M + H-H_2_O, M + H	[82]
105	19.36	Genkwanin	C_16_H_12_O_5_	283.0612	283.0612	M-H	[36]
106	19.37	Casticin	C_19_H_18_O_8_	375.1065	375.1075	M + H	[83]
107	19.37	Acacetin	C_16_H_12_O_5_	285.0748	285.0758	M + H	[84]
108	19.41	Galangin	C_15_H_10_O_5_	269.0455	269.0455	M-H	[85]
109	19.45	Bilobetin	C_31_H_20_O_10_	551.0988	551.0984	M-H	[36]
110	19.47	Kaempferide	C_16_H_12_O_6_	301.0697	301.0707	M + H	[86]
111	19.52	Nobiletin	C_21_H_22_O_8_	403.1378	403.1388	M + H	[81]
112	19.67	Pectolinarigenin	C_17_H_14_O_6_	315.0853	315.0863	M + H	[75]
113	20.44	Isoginkgetin	C_32_H_22_O_10_	567.1270	567.1286	M + H, 2 M + H	[36]
114	21.89	Ruvoside deglycosylation	C_23_H_34_O_5_	389.2336	389.2333	M-H	[36]
115	22.05	Sciadopitysin	C_33_H_24_O_10_	581.1429	581.1443	M + H, 2 M + H	[36]
116	22.69	6-tridecylresorcylic acid	C_20_H_32_O_4_	319.2258	319.2268	M + H-H_2_O, M + H	[87]
117	22.90	Hydroginkgolinic acid	C_21_H_34_O_3_	333.2437	333.2435	M-H	[87]
118	23.92	Bilobol	C_21_H_34_O_2_	317.2485	317.2486	M-H	[88]
119	24.08	Scillaren a deglycosylation	C_24_H_32_O_4_	385.2363	385.2374	M + H	[48]
120	25.18	Anacardic acid d	C_22_H_30_O_3_	341.2121	341.2122	M-H	[87]
120	25.85	Anacardic acid c	C_22_H_32_O_3_	343.2277	343.2278	M-H	[89]
122	26.42	6-tridecylsalicylic acid	C_20_H_32_O_3_	319.2277	319.2278	M-H	[90]
123	26.46	6-[(8z)-pentadecenyl]-salicylic acid	C_22_H_34_O_3_	347.2577	347.2581	M + H-H_2_O, M + H	[86]

## Data Availability

The original data presented in this research are included in the article. Further inquiries can be directed to the corresponding authors.
